# HLA Expression in Uveal Melanoma: An Indicator of Malignancy and a Modifiable Immunological Target

**DOI:** 10.3390/cancers11081132

**Published:** 2019-08-07

**Authors:** Zahra Souri, Annemijn P.A. Wierenga, Arend Mulder, Aart G. Jochemsen, Martine J. Jager

**Affiliations:** 1 Department of Ophthalmology, Leiden University Medical Center (LUMC), Albinusdreef 2, 2333 ZA Leiden, The Netherlands; 2 Department of Immunohaematology and Blood Transfusion, Leiden University Medical Center (LUMC), 2333 ZA Leiden, The Netherlands; 3 Department of Cell and Chemical Biology, Leiden University Medical Center (LUMC), 2333 ZA Leiden, The Netherlands

**Keywords:** uveal melanoma, inflammation, HLA Class I, HLA Class II, chromosome 3, BAP1

## Abstract

Uveal melanoma (UM) is the most common primary intraocular malignancy in adults, and gives rise to metastases in 50% of cases. The presence of an inflammatory phenotype is a well-known risk factor for the development of metastases. This inflammatory phenotype is characterized by the presence of high numbers of lymphocytes and macrophages, and a high expression of the HLA Class I and II antigens. An abnormal expression of HLA Class I may influence cytotoxic T lymphocyte (CTL) as well as Natural Killer (NK) cell responses. We provide a comprehensive review regarding the inflammatory phenotype in UM and the expression of locus- and allele-specific HLA Class I and of Class II antigens in primary UM and its metastases. Furthermore, we describe the known regulators and the role of genetics (especially chromosome 3 and BRCA-Associated Protein 1 (BAP1 status)), and, last but not least, the effect of putative therapeutic treatments on HLA expression.

## 1. Introduction

Uveal melanoma (UM) is an intraocular tumor which arises from the uveal tract, with 3–5% of tumors involving the iris, 5–8% the ciliary body and 90% the choroid [1,2]. The disease occurs mainly in Caucasians, where it is associated with blond hair, light eyes and a fair skin [3,4]. Depending on the size and location of the tumor, the clinical presentation differs. Symptoms may include loss of vision and pain, or the tumor may be found by chance during a routine eye examination [5,6]. The diagnosis is made upon clinical examination by an ophthalmologist using indirect ophthalmoscopy, ultrasonography and sometimes fluorescein angiography. Treatment consists of irradiation (local brachytherapy, proton beam therapy, stereotactic irradiation), or enucleation of the eye. Histopathological examination of tumor material is performed for confirmation of the diagnosis as well as for determining prognostic parameters [6,7,8].

The hematogenous spread of UM specifically targets the liver and up to 50% of patients ultimately die due to metastatic disease [9]. One of the hallmarks of cancer is inflammation [10,11,12]: inflammation stimulates tumor cell proliferation, survival, angiogenesis, metastasis formation, and may cause a decreased response to treatments. Although the eye is an immune-privileged site, where inflammatory responses are limited [13], immune cell infiltration frequently occurs in the intraocular UM [14]. During the course of malignant progression of the tumor, an increase in infiltrating immune cells (high numbers of infiltrating tumor-associated macrophages—TAM’s—and tumor-infiltrating leukocytes—TIL’s—and HLA Class I expression) is observed [15], which combination is known as the inflammatory phenotype [16,17,18,19].

This inflammatory phenotype has been identified as being associated with an infaust prognosis. It is associated with loss of one chromosome 3 and loss of expression of BRCA-Associated Protein 1 (BAP1), the product of a gene located on chromosome 3, which encodes a ubiquitin protease [20]. Loss of expression of BAP1 and loss of one chromosome 3 often occur together and both are well-known risk factors for the development of metastases in UM patients [21,22]. 

**Figure 1:**
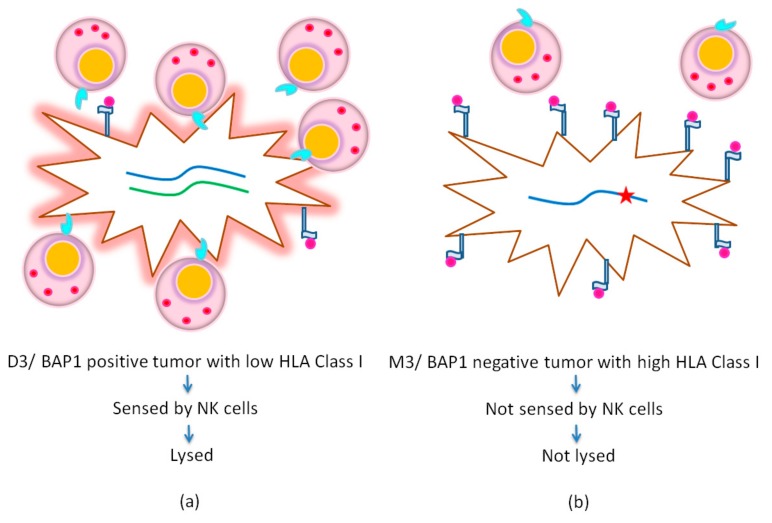
Schematic illustration of the relationship between the tumor’s chromosome 3/BRCA-Associated Protein 1 (BAP1) status, its HLA expression and Natural Killer (NK) cell recognition. (**a**) Disomy 3/BAP1-wildtype tumor: low HLA Class I expression, killed by NK cells. (**b**) Monosomy 3/BAP1-mutated tumor: high HLA Class I expression, escapes from NK cell-mediated lysis in the bloodstream.

HLA molecules are glycoproteins expressed on the cell surface. The *HLA* genes are located on chromosome 6. HLA Class I proteins are present on almost all nucleated cells and serve to present foreign peptides to cytotoxic T cells (CTLs, CD8^+^) or bind to killer inhibitory receptors (KIR’s) of Natural Killer (NK) cells, which may lead to suppression of these NK cells (Figure 1). HLA Class II proteins are mainly expressed on B cells, a subset of T cells, and on antigen-presenting cells and interact with regulatory Treg cells (CD4^+^) [23]. As HLA expression is known as one of the prognostic factors in UM while it is also important for the effectiveness of immunotherapeutic approaches, we here provide an overview of the expression of these molecules in UM, their regulation, and function.

## 2. HLA Expression in Uveal Melanoma 

### 2.1. Variability of HLA Expression in UM

Studies in the 1980s on cutaneous melanoma showed that specific expression patterns of HLA Class I and HLA Class II might be associated with progression to metastasis. HLA Class I, beta-2 microglobulin (B2M) and HLA Class II were not detected on the majority of nevus cells but were found on primary cutaneous melanomas and metastatic lesions [24]. The difference in the expression of these molecules suggested a role in the progression of malignancy. This led to a study on HLA expression in UM, which examined the expression of monomorphic and locus-specific HLA Class I and II antigens on paraffin sections of 27 human UM, using a panel of monoclonal antibodies directed against B2M, HLA Class I and HLA Class II [25]. HLA Class I antigens were expressed on most tumors and more cells expressed Class I than Class II. HLA Class I expression was higher on (high risk) epithelioid tumors than on tumors with a spindle or mixed cell type. There was a great variability in HLA expression among the tumors which theoretically could be caused by different developmental states, different oncogenic mutations and also different lymphokines present in the tumor microenvironment. In 1996, De Waard-Siebinga et al. [26] compared the expression of HLA-A versus HLA-B on paraffin sections of 23 HLA-typed UM and found that HLA-A expression was higher than HLA-B expression. 

### 2.2. HLA Expression and Metastatic Potential

The studies by Jager et al. in 1986 [25] and De Waard-Siebinga et al. [26] in 1996 lacked proper information on the relation with survival. To answer the question whether HLA expression is related to survival, Blom et al. [27] set out to determine the expression of locus-specific HLA-A and HLA-B expression using immunohistochemistry on paraffin sections of 30 UM with good clinical information and excellent follow-up. Confirming earlier findings, they found variability among different tumors for HLA-A and HLA-B expression. However, within individual tumors, HLA-A and HLA-B expression were highly correlated. While the paradigm and expectation was that a low HLA expression would allow tumor cells to escape from CTL-mediated lysis, and thus lead to metastases, this study showed for the first time that the opposite was true: in UM, a high HLA-A/B expression was associated with a low patient survival. The assumption arose that a decreased expression of HLA-A and HLA-B could prevent the development of metastasis because of NK cell-mediated lysis of migrating tumor cells in the blood, preventing the tumor cells from reaching the liver. An earlier study by Ma and Niederkorn in 1995 [28] had already indicated the possible role of NK cell-mediated lysis against UM cells. The group of Niederkorn tested Transforming Growth Factor beta (TGF-β), a down-regulator of HLA Class I, on two melanoma cell lines (OCM1, OCM8) which were known to have high levels of HLA Class I, in order to investigate the effect of NK-mediated lysis. Incubation with TGF-β resulted in a significant downregulation of HLA Class I (52–62%) and an increase of NK cell cytolysis. As an analogous experiment they applied Interferon gamma (IFNγ), an inducer of HLA Class I, on cell line OCM3, which had a low HLA Class I level. This led to an 80% increase in HLA Class I expression and a 10% reduction in NK cell-mediated lysis. Taken together these results confirmed the strong role of different external factors in the tumor microenvironment which could influence the survival and metastasis of melanoma cells, and the role of HLA expression in the effector function of immune cells. The hypothesis became that NK cells in the blood would kill UM cells with a low HLA Class I expression prior to their settlement in the liver, while the HLA Class I expressing cells would not be lysed. These studies stimulated an interest in the function of HLA in UM.

A few years later, papers from three different countries confirmed the findings of Blom et al. [27]. A study by Ericsson et al. [29] not only confirmed the findings with regard to HLA Class I, but also provided information about the association of HLA Class II expression and the progression of the disease in 70 patients. A low expression of HLA Class I and HLA Class II was significantly more frequent in more benign spindle cell UM (*p* = 0.006 and *p* = 0.01, respectively). A high expression of HLA Class I, B2M and HLA Class II correlated significantly with the development of metastases (*p* = 0.013, *p* = 0.001, and *p* = 0.02, respectively). These findings support the role of NK cell-mediated protection against systemic spreading of UM. In 2002, Dithmar et al. [30] looked at the association between HLA Class I locus-specific expression and melanoma cell type: 22 tumors were divided into those with a spindle or those with an epithelioid cell type. HLA Class I expression was analyzed by immunohistochemistry, using the same antibodies as used previously by De Waard-Siebinga et al. [26] and Blom et al. [27]. 18% of the spindle type UM versus 82% of the epithelioid type UM stained positively with (HCA)-10, an antibody that recognizes specifically HLA-A antigens [31]. They confirmed that the more malignant epithelioid cell type tumors had a higher HLA expression than non-epithelioid tumors.

### 2.3. Coordination between HLA Expression and Tumor Infiltrates 

An early study in 1992 used monoclonal antibodies and flow cytometry to study the lymphocyte and monocyte population and HLA expression in 41 UM samples [32]. Meecham et al. determined the presence of melanoma cells using antibody 13A3E (an anti-melanocyte antibody) which stained on average 82% of the cells (31–98%). Staining for HLA-A, HLA-B and HLA-C was positive in on average 85% of the tumor cells (25–98%) and for the Class II antigen HLA-DR in 7% (0–58%). The amount of lymphocytic infiltrate was variable among the samples with predominantly CD3^+^ T cells, accounting for 4.5% of the total cell population (range 0.1–29%). Other immune cells such as NK cells, B cells and macrophages comprised less than 2.5%. They found that the ratio of the CD4^+^/CD8^+^ population was not constant, as in some tumors the CD4^+^ population was dominant while in others CD8^+^ T cells were the most common. This study showed that (in irradiated tumors), an increase in age was associated with an increase in the CD4^+^ population, while the number of CD8^+^ cells decreased (*p* = 0.02). In 1996 [14], De Waard-Siebinga et al. found significant positive correlations between the presence of CD3^+^ cells and HLA Class I, HLA-A2, HLA-Bw4 and HLA Class II expression. In addition, CD4^+^ T cells and CD11B+ cells (granulocytes, monocytes/macrophages, NK cells) were significantly correlated to HLA class I expression. All UM samples contained some infiltrating cells although often in small amounts, with a predominance of T cells.

Van Essen et al. [33] compared the presence of molecules of the Antigen Processing Machinery (APM) with the quantity of infiltrating cells and similarly observed that a high number of macrophages (CD68^+^) and lymphocytes (CD3^+^) correlated positively to the level of HLA Class I expression as they did with the status of chromosome 3. *HLA-A*, *HLA-B*, and *B2M* were all higher in tumors with a high CD3^+^ infiltrate. When fresh tumor material was implanted as a xenograft into Severe Combined Immunodeficient (SCID) mice, leukocyte infiltration was lost and subsequently, the expression of HLA Class I and its regulators became downregulated. These findings show that it is the presence of leukocytes that causes the upregulation of HLA Class I and II antigens, and that is not the high level of HLA antigens attracting the infiltrating immune cells. 

### 2.4. Locus and Allele-Specific Loss as an Escape Strategy

**Figure 2:**
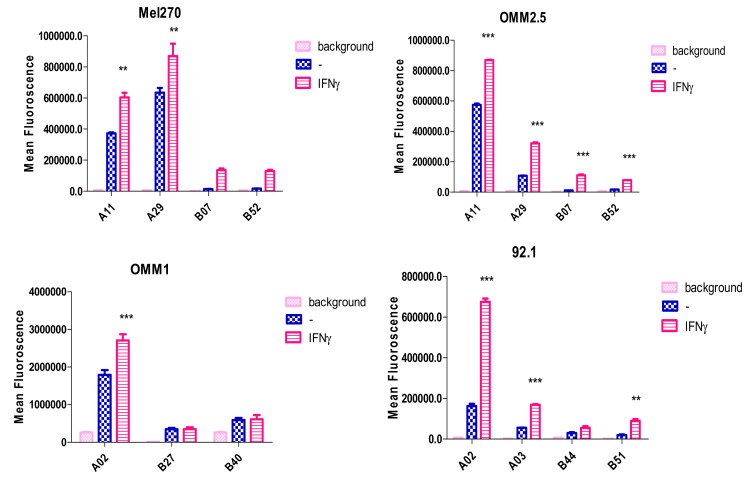
HLA-typed Uveal melanoma (UM) cell lines can be analysed by Fluorescence-Activated Cell Sorting (FACS) using HLA antigen specific monoclonal antibodies to find defects in the expression of HLA alleles, and to determine the sensitivity of induction by Interferon gamma (IFNγ). Loss of HLA Class I allele expression in UM cell lines: B7 and B52 loss in Mel270, B27 and B40 loss in OMM1, B44 loss in 92.1. (** *p* = 0.01; *** *p* = 0.001).

During the last decades, several studies have confirmed that UM cells may lack expression of locus or allele-specific HLA antigens, which may help cells to escape from T cell-mediated responses while the normal expression of the remaining antigens would give the cells the ability to be protected against NK cells inside the bloodstream. In 2002, Anastassiou et al. [34] studied HLA-A, HLA-B, and HLA-C expression using immunoprecipitation and Western blot on tissues of 18 HLA-typed patients. Half of the samples showed full HLA-A and HLA-B expression, while HLA allotype loss was found in 33%, with three cases affecting the HLA-A locus (HLA-A2, A28, and A29) and three the HLA-B locus (HLA-B18, B35 and B55). Two tumors showed a haplotype loss (HLA-A2, B44 and HLA-A2, B13) and one tumor showed a complete HLA-A (HLA-A26, -A32) loss together with one HLA-B allotype loss (HLA-B41). HLA-A2 expression was variable, with homogenous expression in three samples (>75% of the tumor cells), heterogeneous expression in two samples (25–75% of the tumor cells) and negative expression in only one sample. The cause for lack of expression was not investigated. In order to find out whether immunotherapy would be feasible on UM, our lab studied the reason why specific T cells would not recognize tumor cells in spite of high levels of HLA Class I expression in UM [35]. It was suggested that there might be an association between loss of specific HLA haplotypes and T cell recognition. A series of molecularly HLA-typed cell lines (92.1, Mel202, OCM-1, EOM3, OCM-3 and OMM1) was tested using Fluorescence-Activated Cell Sorting (FACS) in order to find defects in polymorphic HLA-A and HLA-B expression. HLA-A expression was high in all the tested cell lines and could be further (and similarly) induced by both IFNγ and IFNα treatment [36,37]. Both allele-specific and locus-specific loss of HLA expression were observed in UM cell lines, and some could be restored by IFN treatment, others could not. Cell line 92.1 had lost HLA-B44, and Mel202 had lost its HLA-B5-allele specific expression while OMM1 had lost all B locus-specific expression (HLA-B27, -B40). This loss of expression may have helped the tumor to prevent recognition by CTLs, helping metastasis to develop [16]. Recently, we tested four UM cell lines and used IFNγ to induce HLA Class I expression (Figure 2).

## 3. HLA Class I Regulation in UM

### 3.1. HLA Expression is Related to Genetic Factors

In other tumors such as cutaenous melanoma, several different mechanisms have been found responsible for a loss in HLA antigen expression. We asked the question whether genetic determinants influenced the level of expression of HLA. Van Essen et al. [38] compared HLA expression with the distribution of the HLA allele frequencies in 50 patients who had undergone enucleation for UM in Leiden between 1999–2004. Before correction for multiple testing, a lower macrophage infiltration was seen for tumors that were HLA-A2, and a higher HLA-DR expression in tumors of patients with HLA-DR6 patients, although after correction for the number of analyses, no significance was found. This study showed that the HLA genotype does not affect overall HLA expression or macrophage infiltration in UM.

An inverse correlation between HLA Class I expression and expression of a well-known oncogene, c-myc, has been observed in cutaneous melanoma [39]. In 1997, Blom et al. asked whether they could find a similar inverse association in UM, and studied paraffin sections from 30 UM, where they measured HLA-A, HLA-B and c-myc expression by immunohistochemistry [40]: a low level of HLA-B was significantly correlated to a high level of c-myc expression in the cytoplasm (*p* = 0.03), which was similar to the findings that had been reported regarding cutaneous melanoma. In the UM sections, a high level of HLA-B was associated with an epithelioid cell type (*p* = 0.004). In other malignancies, loss of HLA Class I antigens is often caused by loss of essential molecules of the HLA antigen-processing and presentation system, such as the Transporter Associated with Antigen Processing (TAP1) protein in colorectal cancer [41,42] and primary cutaneous melanoma [43,44]. Studies were undertaken to determine whether the same held true for UM. 

A study in 2003 assessed the relation between HLA Class I and the presence of Antigen-Processing Molecules (APM) in 41 primary UM specimens from Asian-Indians [45]. HLA Class I, Low Molecular Mass Polypeptide 2 (LMP2), LMP10, TAP1, tapasin and calnexin were all low in tumors without extra-scleral extension and high in tumors which gave rise to liver metastasis. Later, van Essen [33] not only analyzed peptide-loading components but also some other regulators of expression, and (similarly) found significant associations between the presence of *TAP1* and *TAP2* and expression of *HLA Class I*, *HLA Class II* and *B2M*. In addition, the HLA expression regulators Interferon Regulatory Factor 1 (*IRF1*) and *IRF8* were found to correlate positively with *HLA-A, HLA-B* and *B2M*. Regulator NOD-like Receptor family CARD domain containing 5 (*NLRC5*) correlated with *HLA-B* and *B2M* expression, and Class II major histocompatibility complex Transactivator (*CIITA*) with *HLA-B* as well as with *B2M*. This study compared expression with the most important genetic prognostic factor, monosomy for chromosome 3. The relation between *HLA-A* and *HLA-B* expression with chromosome 3 status was investigated by Illumina microarray, immunohistochemistry and qPCR. Illumina data showed a strong difference between disomy 3 (D3) and monosomy 3 (M3) for *HLA-A* and *HLA-B*, with a higher expression in M3 tumors. Immunohistochemistry revealed only a difference for HLA-A, while qPCR revealed differences for HLA-A, HLA-B and B2M between D3 and M3 tumors. While an increased level of *IRF1* was associated with M3, expression levels of *IRF2*, *IRF8*, *NLRC5*, and *CIITA* were not significantly related to M3. However, the group of analyzed tumors was quite small (*n* = 13). A high expression of *TAP1* and *IRF1* was associated with death due to metastases, while *NLRC5*, *CIITA*, *IRF2*, and *IRF8* expression levels were not. This study confirmed that UM cells contain a proper functioning HLA antigen-processing system which therefore do not cause loss of HLA expression, and that expression of HLA proteins on the cell surface, and several HLA transcription regulators and the peptide loading machinery are co-regulated.

### 3.2. Locus-Specific Differences between HLA-A and HLA-B

Low constitutive HLA Class I expression is observed among different types of cells with some being restored by the addition of IFN’s and tumor necrosis factor (TNF). In 1993, Girdlestone et al. investigated the reason for a different regulation of HLA-A and HLA-B [46]. They found a role for Nuclear Factor kappa-light-chain-enhancer of activated B cells (NFkB) in the regulation of HLA-A and HLA-B expression in MOLT4 and YHHH cell lines, which had been derived from lymphoblastic leukemia. Two main upstream control elements are known to be responsible for basal HLA Class I expression. The HLA-A enhancer contains two Rel (kBF/NFkB) binding motifs while the HLA-B enhancer has only one and transcription of the *HLA-B* gene is therefore less trans-activated by the NFkB p65 subunit. In contrast, *HLA-B* transcription was stronger induced by IFNγ than *HLA-A* transcription and the *HLA-B* promoter was found to have a higher affinity for the *IRF1* and *IRF2* transcription regulators. They also found that in cutaneous melanoma, IRF1 binds to HLA-A Interferon Response Element (IRE) with a lower affinity than to the *HLA-B IRE*, explaining why *HLA-A* and *B* genes are distinctly transcriptionally regulated by Rel family members and IFN’s. Also in another study, Johnson et al. reported that HLA-A, B and C differ in their regulation by cytokines and expression in different tissues [47]. 

## 4. Other Types of HLA Class I in UM

In addition to HLA-A and -B, several other Class I molecules can be expressed, with different functions. HLA-C is a member of the HLA Class I family with a relatively low cell surface expression. The main function of HLA-C is its ability to bind as a ligand to KIRs of the NK cells and suppress their cytotoxicity [48]. HLA-G together with HLA-E are members of the non-classic HLA Class I family expressed mainly on fetal cells of human placenta. These molecules are known to have the ability to suppress immune cell functions such as NK and CTL-mediated cytolysis [49,50,51]. HLA-G has so far not been detected in UM [52]: a study in 2002 used a variety of methods to analyse HLA-G expression in 11 human UM cell lines with different HLA-A and -B expression levels and metastatic potential. In addition, HLA-G expression was investigated on 17 frozen primary UM sections. The trophoblast cell line JEG-3 was used as a control. No HLA-G protein or RNA was found in any UM cell line or tissue even after treatment with IFNγ. Different levels of HLA-E expression were observed, which could be further induced after IFNγ treatment, suggesting that HLA-G has no role in a tumor’s escape from the immune system, while HLA-E should be further investigated. As the previous studies demonstrated a low expression of HLA-G in UM it is conceivable that NK cell-based lysis may be effective in destroying UM cells and that these molecules are not involved in the immune-biology of UM.

## 5. Genetics Play a Role in the Development of UM

### 5.1. HLA Allele Frequencies in UM

Several investigators in Leiden studied the genetic distribution of *HLA Class I*, *II* and *MICA* genes in UM patients. In 2005, Metzelaar-Blok et al. analyzed 159 cases of UM which were typed for *HLA Class I* and *II* and 168 cases which were evaluated for the *MICA* gene by microsatellite typing, and compared their HLA genotypes to 2440 healthy controls [53]. No significant differences were observed between the two populations. In the UM population, the highest allele frequency was seen for *A2* (55% in UM and 53% in control population), a gene that is often used for inclusion in immunotherapy trials. No significant associations were noticed between *HLA* and *MICA* genetic polymorphisms and the development of a UM. An extension of this study to 235 cases did not change the conclusions [54], but did find some associations between tumor characteristics (*HLA-DR13* with large tumor size, *HLA-B35* with spindle cell type and *HLA-B60* with ciliary body involvement). While the first study on the relation between HLA type and prognosis showed an association between the presence of *HLA-B40* and the development of metastases [55], the later study by Maat in 2006 did not reproduce this, but rather found an association between *B44* and a worse survival [54].

### 5.2. Somatic Genetic Abnormalities and HLA Expression in Uveal Melanoma

**Figure 3:**
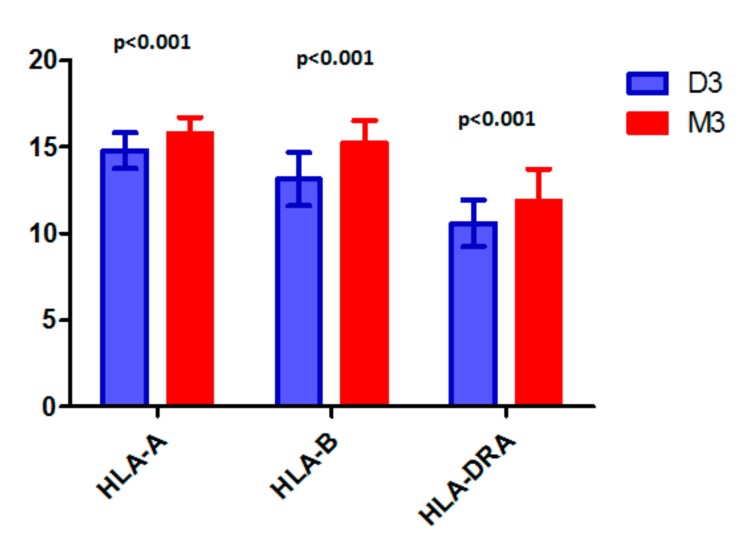
Comparison between HLA-A, HLA-B and HLA-DR expression in disomy 3 (D3) vs monosomy 3 (M3) tumors of The Cancer Genome Atlas (TCGA) database.

Somatic genetic factors are known to influence the development and behavior of UM, and have been found to play a major role in creating the inflammatory microenvironment. Loss of chromosome 1p, 3, or 6q and gain in 6p or 8q are among the most frequently-occurring chromosomal changes identified in UM. These genetic abnormalities affect different aspects of UM [7,8,56,57,58]. Loss of one chromosome 3 (Monosomy 3, M3) is found in 50% of UM patients and is associated with a bad prognosis. Almost all M3 tumors also show gain in chromosome 8q, while this aberration can also be present in Disomy 3 (D3) tumors [59,60,61]. Gain of chromosome 8q is considered an early event, developing prior to the loss of chromosome 3, is a bad prognostic factor and is associated with an increased macrophage infiltration [15]. As the *HLA* genes are located on chromosome 6 and both losses and gains of parts of this chromosome are common in UM, the relation between these genetic aberrations and HLA expression was investigated by several authors. Blok et al. used three microsatellite markers on chromosome 6 to see whether Loss of Heterozygozity (LOH) of chromosome 6p might affect HLA Class I expression [62]. She analyzed DNA extracted from 20 formalin-fixed paraffin-embedded primary UM, and found that 65% of the studied tumors showed LOH of at least one locus on chromosome 6p. However, no correlation between LOH on 6p and HLA-A and HLA-B monomorphic expression was observed. Van Essen et al. [33] investigated putative associations between copy numbers of multiple chromosomes and *HLA* expression. Half of the studied UM showed M3 according to Single Nucleotide Polymorphism (SNP) analysis, which was associated with death due to metastasis (Kaplan-Meier, *p* < 0.001). Gain in 6p was reported in 29% and was associated with good survival (Kaplan-Meier: *p* = 0.049). M3 was associated with elevated levels of *HLA Class I* and *B2M*. When looking at all tumors together, an association was observed between 6p gain and a low *HLA-B* expression (*p* = 0.049), which was probably due to the negative association between M3 and 6p gain: among the D3 tumors, no dosage effect of 6p was found on *HLA Class I*, *II* or *B2M* gene expression while M3 tumors had higher mean level of *HLA Class I* compared to D3 tumors. As shown in Figure 3, HLA-A, HLA-B and HLA-DR are significantly higher in M3 tumors compared to D3 tumors according to data analysed from The Cancer Genome Atlas (TCGA) database [18]. 

## 6. Metastases and HLA Expression

When comparing expression of monomorphic HLA Class I on a primary UM sample, and several metastases, Blom et al. [63] noticed that in the metastatic lesions, expression of polymorphic HLA-A2 and HLA-A3 was decreased. Changes in HLA-B expression could not be assessed as HLA-Bw4 was low in all lesions. All metastases contained high amounts of CD3^+^ and CD4^+^ and a lower amount of CD8^+^ cells. The tissue of the metastasis was epitheloid, which was the same as the primary tissue but with less coherence and atypical mitoses. Verbik et al. [64] compared the ability of T cell stimulation in primary melanoma cell lines derived from either eye or skin melanoma. The expression of HLA Class I and II was assessed either by absence or presence of IFNγ. Also, various concentrations of tumor cells were added to lymphocyte cultures and their stimulatory capacity on T cells was determined. Primary cutaneous melanoma cells induced T cell proliferation while UM cells did not, although the UM cells expressed high levels of HLA Class I and II after stimulation with IFNγ. The inhibitory effect was lost when ocular cells formed hepatic metastasis. This suggested that primary ocular tumor cells were poorly immunogenic and this immunogenicity alters when they move to a new microenvironment. In 2017, Gezgin et al. reported on a series of metastases [65]. Previously, Melanoma Antigen Preferentially Expressed in Tumors (PRAME) was found to be expressed on primary UM [66]. Gezgin observed that PRAME is indeed expressed in almost half of UM cases and is associated with largest basal diameter (15 mm vs 12 mm *p* = 0.005), ciliary body involvement (59% vs 26%, *p* = 0.008) and gain of chromosome 8 (66% vs 23%, *p* = 0.002). PRAME-specific T cells reacted with four out of seven UM cell lines. Of UM metastases, 69% were positive for PRAME mRNA, and 63% positive for HLA Class I, with a total of 50% samples co-expressing HLA and PRAME. This study confirmed that PRAME is expressed in UM and that some metastatic samples co-express HLA and PRAME, which makes the PRAME antigen a putative target for PRAME-directed immunotherapy.

In order to study changes during tumor progression and discover potential pathways in the development of metastasis in UM, Meir et al. [67] analyzed seven metastases and seven primary UM by microarray analysis, validating their study by qPCR and immunohistochemistry. Microarray analysis showed that 193 genes were differently expressed between metastasis and primary UM, with 184 increased in the metastasis. NFkB2 was increased in the metastasis, with an increased expression of downstream genes which are involved in the progression of the disease such as Growth Arrest and DNA-Damage-inducible, Beta (GADD45B) and Hedgehog Interacting Protein (HHIP).

## 7. The Effect of Different Treatments on HLA Expression in UM

### Interferon Induces HLA Expression

In order to achieve an effective immune response against tumor cells it is crucial that tumor antigens get displayed on the cell surface by sufficient numbers of HLA Class I molecules. The cytokine IFNγ has the ability to up-regulate HLA expression on UM cell lines in vitro [36], which could suggest that environmental factors may influence HLA expression as well. De Waard-Siebinga et al. [68] compared HLA expression in short term UM cultures with the expression in the original tumors from which the cultures had been derived. Immunohistochemistry was used to measure HLA expression on tissue sections (HCA2, HC10 and allele-specific B8.11.2, BB7.2, GAP.A3, 116/5/28, SFR-8-B6) and monoclonal antibodies W6/32 and BBM1 were used to evaluate HLA Class I expression on cultured cells. HLA-A expression in the cultured UM cells correlated to the tissue expression (R = 0.77) while HLA-B was less correlated (R = 0.68). HLA-DR expression was decreased during culture, probably because the new microenvironment of the cells did not contain interferon-producing leukocytes. In a later study, De Waard-Siebinga et al. [69] established UM cell line 92.1 at the Leiden University Medical Center. Immunohistochemistry, flow cytometry and Northern blot analysis for HLA Class I revealed that this cell line had a low amount of HLA-B compared to HLA-A which, however, could be further induced by IFNγ. The effect of IFNγ and IFNα on the growth and expression of HLA Class I and II was studied on cell lines 92.1 and Mel202 by cytospins and flow cytometry [36]. Treatment with either IFNγ or IFNα decreased the growth of 92.1 but only IFNγ was able to inhibit the growth of Mel202. IFNα increased only HLA Class I on the two cell lines. At that time the exact mechanism of action of IFN on the cell lines was not well known but as differences were observed with regard to the induction of HLA among cell lines, this study reflected a potential individual difference in response to treatment with interferon’s in vivo.

**Figure 4:**
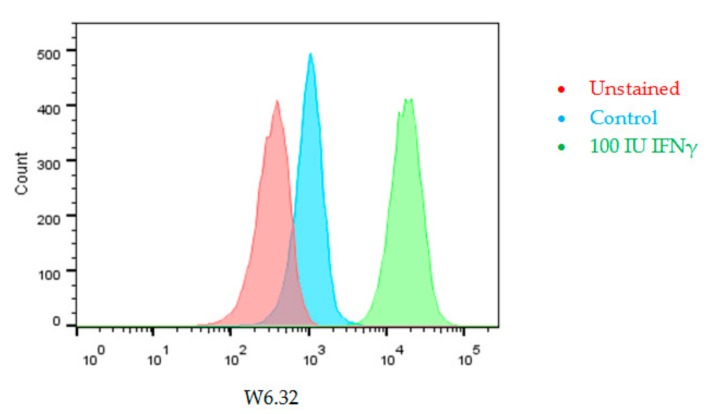
Interferon gamma (IFNγ) treatment induces HLA Class I expression, determined with monoclonal antibody W6/32 and Fluorescence-Activated Cell Sorting (FACS) analysis on Uveal melanoma (UM) cell line 92.1, after 48 h treatment.

We investigated the expression of HLA Class I (using monoclonal antibody W6/32) on UM cell line 92.1 (Figure 4) and show by FACS that IFNγ induces HLA Class I expression. 

## 8. HLA Class II in UM

Under normal physiological conditions, constitutive HLA Class II expression mainly occurs on the surface of antigen-presenting cells and thymic epithelial cells [70]. Krishnakumar et al. analysed HLA Class II expression on 45 primary UM from Asian-Indian patients [71], using the anti-HLA Class II mAb LGII 612.14. In their study, 17 of the tumors were spindle cell type, 16 mixed cell type and 12 epithelioid cell type. Thirty-five UM had no extrascleral extension and showed a low HLA Class II expression. Among the remaining 10 melanomas with extrascleral extension, 60% had developed metastases and revealed a high HLA Class II expression, while 40% did not develop metastases and had a low HLA Class II expression (*p* ˂ 0.001). 

### 8.1. Irradiation and HLA Class II Expression

In 1988, it was shown that expression of HLA-DQ was correlated with the presence of a lymphocyte infiltrate, suggesting a potential regulatory relationship between HLA Class II and infiltrates [72]. Comparing non-irradiated tumors with irradiated ones showed differences in all three HLA Class II antigens: DR, DP and DQ, although the level of HLA Class I, B2M and various melanoma-associated antigens remained the same. This suggested that irradiation might directly influence the tumor cells or indirectly influence HLA Class II expression via alteration in the extent of the infiltrate, suggesting that radiotherapy decreases the amount of lymphocytic infiltration: less IFN is then produced that could upregulate HLA Class II expression. Ericsson et al. [29] found that HLA Class II expression was expressed on 30 out of 65 UM samples and expression was associated with a lower survival. This study found higher levels of HLA Class II expression than the study of Jager in 1988 and it was suggested that in the previous study, irradiation might have decreased the expression.

### 8.2. Regulation of HLA Class II

Radosevich et al. found that UM cells have resistance to IFN induction of HLA Class II [73], but that this was not due to blocking of the biosynthetic pathway or the IFNγ signal transduction pathway. They used the Mel202, Mel270 and 92.1 cell lines, with Jurkat cells as control and found that, similar to other reports, *CIITA* expression was decreased in the UM cell lines. After addition of the DNA methylation inhibitor 5-aza-2’-deoxycytidine, the cells again expressed *CIITA* mRNA and also *HLA-DRA* mRNA. They therefore concluded that DNA methylation is a strategy for the tumor cells to maintain themselves in the immune-privileged eye, by downregulating their HLA Class II expression. 

When examining leukemic T cells, Holling et al. found that *CIITA* gene expression is silenced by an epigenetic mechanism rather than lack of transcription factors [74]. They subsequently investigated melanoma cell lines (Mel285, OMM1.3, OCM-1, OCM-3) [75] and observed a lack of response to IFNγ induction when there were high levels of DNA methylation of the MHC2TA promotor IV (CIITA-pIV), and with high levels of tri-methylated histone H3-lysine 27. This resulted in low *CIITA* and *HLA Class II* expression. Histone methyltransferase EZH2 (Enhancer of Zeste Homolog 2) contributed to this silencing of IFN γ-inducible transcription of *CIITA*. Boyd et al. described the role of two other DNA-binding proteins that interact with *CIITA* pIV, namely Yin Yang 1 (YY1) and Jumonji domain containing protein 2 (JARID2) [76]. They show that these proteins are involved in recruitment of the silencing complex Polycomb Repressive Complex 2 (*PRC2*) (which contains EZH2) to the pIV promoter. Jumonji domain containing protein 2 (*JARID2*) knockdown resulted in elevated levels of *CIITA* mRNA upon IFNγ stimulation, suggesting that *JARID2* is an inhibitor of HLA Class II activation. Interestingly, other studies have also described a role for Yin Yang 1 (*YY1*) in regulating HLA [77,78].

## 9. Environmental Influences

Several studies have analyzed environmental influences, such as photodynamic therapy and hyperthermia, on expression of HLA in cultured UM cells. An early study [32] analyzed tumors that had previously been treated with either helium ions or I125 plaque. These tumors had a reduced staining with anti-melanoma antibody 13A3E because of tumor cell destruction or showed an altered antigen expression. No clear relationship was observed between HLA Class I expression and cell type. Blom et al. evaluated the effect of photodynamic therapy (PDT), using the hematoporphyrin ester bacteriochlorin on UM cell line 92.1 [79]. Flow cytometry analyses showed that HLA-A,-B,-C and B2M microglubulin expression was reduced after PDT treatment, increased after 2 h and normalized after 6 h of treatment. Blom et al. similarly tested the effect of hyperthermia on HLA Class I, B2M, Heat Shock Protein (HSP)-60 and HSP-70 [80]. They found a time and temperature-dependent effect of this treatment on HLA Class I and HSP-70: exposure to 45 °C increased HSP-70, but not HSP-60 and reduced HLA Class I expression. However, no effect was observed on NK cell susceptibility.

**Figure 5:**
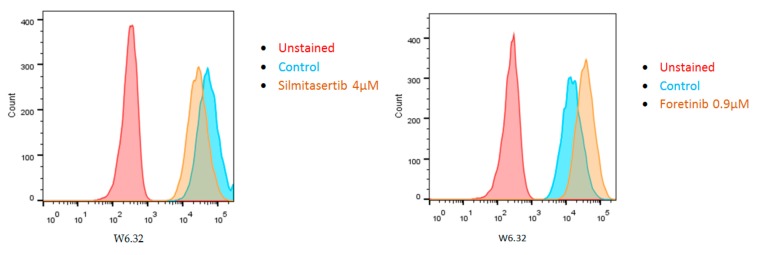
Treatment of cell line OMM2.5 with two different kinase inhibitors for 48 h either reduced (silmitasertib) or increased (foretinib) HLA Class I expression (monoclonal antibody W6/32, measured by Fluorescence-Activated Cell Sorting (FACS) analysis). In the control, no drug was added.

Recently, we determined whether certain therapeutic drugs, which are being tested to treat metastasis of UM, influence HLA Class I expression (Figure 5). Our results show that 48 h treatment with silmitasertib (an inhibitor of Casein Kinase 2 and Clks) reduced HLA Class I expression in OMM2.5 while foretinib (an inhibitor of c-Met, Vascular Endothelial Growth Factor (VEGF) receptor-2 and Tumor Associated Macrophage (TAM) receptors) increased HLA Class I expression. More small molecule compounds are being tested, but these data show it should be realized that (chemo)therapy can influence the expression of immunologically relevant molecules such as the HLA antigens.

## 10. Potential for Immunotherapy: Function of HLA Antigens in Antigen Presentation

Murine experiments demonstrated that UM cells in the eye are potential candidates for T cell mediated therapy [81]. Sutmuller et al. immunized HLA-A*0201/Kb (A2/Kb)-transgenic mice with recombinant canarypox virus (ALVAC-gp100) and isolated HLA-A*0201-restricted CTL against human gp100. After injection of human HLA-A2 positive UM cells into the anterior chamber of A2/Kb-transgenic mice, these CTL’s were injected systemically, which resulted in a rapid elimination of the UM cells from the murine eye. These data show that immunological treatment of intraocular tumors should be possible in spite of the immune privilege.

Luyten et al. studied eight UM cell lines to assess their capacity to be used as stimulators in immunotherapy [82]. The expression of human Melanoma Associated Antigen (*MAGE*)*, -1, -2* and *-3*, *gp100* and *Tyrosinase* was measured in UM cell lines. Cell lines OCM.1 and OMM1 expressed *MAGE-1, -2* and *-3*, whereas EOM-3, Mel202, 92.1, and OMM3 did not. *Gp100* was expressed in all cell lines, while *Tyrosinase* was not expressed in EOM.29, OMM2 and OMM3. They tested the effect of various CTLs in a complement-dependent microlymphocytotoxicity assay and showed the presence of HLA Class I expression on the primary UM cell lines and HLA-A1 or HLA-A2 allelic expression on the metastatic cell lines. Because the cell lines expressed HLA Class I molecules and at least two melanoma-associated antigens, they could be used as targets to improve experimental immunotherapy. Bosch et al. designed Major Histocompatibility Complex II (MHCII)-matched vaccines from UM individuals which could cross react with HLA-DR-restricted Tregs of other UM patients and induce IFNγ secretion in them, allowing for a CD8^+^ cell immune response in the eye [83]. In order to develop MHC II vaccines they used cell lines Mel202, Mel270 and OMM2.3 which express HLA Class I but not Class II or the invariant chain. Expression of HLA-DR and the co-stimulatory molecule CD80 was induced by transduction with retroviruses encoding HLA-DRB1*0101 (DR1) and/or the co-stimulatory molecule CD80. The MHC II generating vaccine cells were able to stimulate T cell responses, and were stable for 6 months in culture.

Indoleamine-2,3-dioxygenase 1 (IDO1) is a potential immune modulator. Mondanelli et al. focused on two arginase 1 (Arg1) enzymes, both of which are involved in the regulation of the immune system [84]. When studying their activity in dendritic cells, they observed that the cytokine TGFβ was able to upregulate Arg1 and IDO1 together, with Arg1 getting upregulated earlier than IDO1. Their study led to their understanding that Arg1 is essential for the activity of IDO1 and both are expressed at the same time in dendritic cells. Li et al. used a special vector to induce IDO expression and observed that IDO suppressed HLA Class I expression in keratinocytes [85]. IDO1 is expressed in primary UM and at low levels also in metastases [86,87]. It would be interesting to see whether induction of IDO could serve as a potential mechanism to downregulate HLA Class I and lower the tumor cell’s metastatic potential in UM. 

## 11. New Technologies Might Serve to Improve Knowledge about HLA Expression in UM

Many of the studies on HLA expression in UM used monoclonal antibodies on fresh-frozen tissue sections and mRNA expression. Nowadays, new techniques have become available that may help to further our knowledge of the immune system and improve cancer therapy. De Lange et al. showed that droplet PCR that identifies DNA differences is a very useful technique to determine tumor heterogeneity; this technique is being used to determine the percentage tumor cells in a sample, as well as the percentage of T and B cells [88,89]. Mass cytometery (CyTOF) is another method which can assess different subpopulations of infiltrating immune cells in small tissue samples [90]. Using this technology, antigen-presenting cells can be categorized into different subsets according to their phenotype and function. Lingblom et al. used this technology to characterize immune cell populations in the blood before and after administration of a vaccine against the respiratory syncytial virus (RSV). They found differences between HLA-DR expression on both CD4^+^ as well as CD8^+^ cells between responders and non responders to the vaccine [91]. 

Other new molecular techniques use genetic and RNA information to precisely obtain information of the genome and transcriptome and classify the characteristics of different diseases. RNAseq analysis of primary UM and its metastases may help to assess the differential expression of different HLA alleles, and its association with different infiltrating leukocyte types, and molecular pathways. Robertson et al. not only used RNA but also microRNA and long non-coding RNAs (lncRNA) to subdivide UM tumors according to their molecular chracteristics in order to better understand how each population’s molecular signiture is related to clinical outcome [18]. However, this study still needed fresh-frozen tumor tissue. A recent study used paraffin-embedded tissues when applying nanostring technology for the analysis of RNA in addition to microarray data in cutaneous melanoma patients who had been treated with anti-PD1. Using this new technology, they found that a special pathway related to hypoxia was activated in hypoxic regions. This pathway induces autophagy and eventualy leads to resistance of the tumor cells against T cell therapy [92]. Recently, hypoxia as defined by expression of Hypoxia-Inducible Factor 1 (HIF1a) was found to be associated with loss of BAP1 expression in UM, and may become a target for therapy [93,94]. Using these new techniques to develop an insight in the pathophysiology of the relation between the function of BAP1, the immune system, and the development of metastasis may help to develop a treatment for UM patients.

## 12. Conclusions

Several studies have been performed on the role of HLA in UM and all confirm that a high HLA Class I expression is associated with a bad prognosis, in contrast to the situation in many other malignancies. A high HLA expression is associated with loss of one chromosome 3/loss of BAP1 expression, and is associated with the presence of infiltrating lymphocytes and macrophages, in spite of the eye being an immune-privileged site. It is likely that a genetically-determined upregulation of HLA antigens attracts leukocytes, which produce IFN, which further stimulates HLA Class I expression [95]. Epigenetic modifications influence expression, and loss of expression of the ubiquitin protease BAP1 is associated with an increased expression of HLA Class I antigens. It is as yet unknown why BAP1 has such a great influence on HLA expression and infiltration of immune cells in UM. Further studies are necessary to determine whether this is also relevant inside metastases. 

The inflammatory microenvironment in the primary tumor creates an opportunity for the UM cells to evade NK cells when they migrate haematogenously from the eye to the liver. As another strategy to avoid recognition from the immune system, UM cells may loose expression of their genetically-determined HLA antigens, with allele-, locus- or haplotype loss, which helps tumor cells to escape from cytotoxic T cells. The cell lines which are currently used in research laboratories represent many of the characteristics of primary and metastatic UM and serve as good and comparable models of UM tumors for future observations, helping to understand the regulation of HLA antigen expression. Cell line studies may be useful to identify the effect of different drugs on the expression of HLA antigens and immunomodulating proteins such as IDO1 and PD-L1. As HLA antigens are necessary for T cell therapy, knowing how they are expressed and regulated helps to understand which patients are good candidates for immunotherapy.
